# Probing the Design Rationale of a High‐Performing Faujasitic Zeotype Engineered to have Hierarchical Porosity and Moderated Acidity

**DOI:** 10.1002/anie.202005108

**Published:** 2020-08-25

**Authors:** Stephanie Chapman, Marina Carravetta, Ivana Miletto, Cara M. Doherty, Hannah Dixon, James D. Taylor, Enrica Gianotti, Jihong Yu, Robert Raja

**Affiliations:** ^1^ School of Chemistry University of Southampton Highfield Campus Southampton SO17 1BJ UK; ^2^ Department of Science and Technological Innovation Università del Piemonte Orientale Viale T. Michel 11 15121 Alessandria Italy; ^3^ CSIRO Manufacturing Private Bag 10 Clayton South Victoria 3169 Australia; ^4^ ISIS Hydrogen and Catalysis Laboratory, ISIS Pulsed Neutron and Muon Facility STFC Rutherford Appleton Laboratory Chilton Didcot OX11 0QX UK; ^5^ State Key Laboratory of Inorganic Synthesis and Preparative Chemistry College of Chemistry International Center of Future Science Jilin University 2699 Qianjin Street Changchun 130012 China

**Keywords:** acid catalysis, heterogeneous catalysis, hierarchical porosity, probe-based techniques, structure–activity relationships

## Abstract

Porosity and acidity are influential properties in the rational design of solid‐acid catalysts. Probing the physicochemical characteristics of an acidic zeotype framework at the molecular level can provide valuable insights in understanding intrinsic reaction pathways, for affording structure–activity relationships. Herein, we employ a variety of probe‐based techniques (including positron annihilation lifetime spectroscopy (PALS), FTIR and solid‐state NMR spectroscopy) to demonstrate how a hierarchical design strategy for a faujasitic (FAU) zeotype (synthesized for the first time, via a soft‐templating approach, with high phase‐purity) can be used to simultaneously modify the porosity and modulate the acidity for an industrially significant catalytic process (Beckmann rearrangement). Detailed characterization of hierarchically porous (HP) SAPO‐37 reveals enhanced mass‐transport characteristics and moderated acidity, which leads to superior catalytic performance and increased resistance to deactivation by coking, compared to its microporous counterpart, further vindicating the interplay between porosity and moderated acidity.

## Graphical abstract

A design‐led dual‐templating approach was used to create a hierarchical zeotype catalyst with superior mass transport properties. Advanced probe‐based characterization (including positron annihilation lifetime spectroscopy and probe‐based IR spectroscopy) shows how the siliceous mesoporogen modulates the porosity and moderates the acidity of the zeotype framework, leading to enhanced catalytic performance.

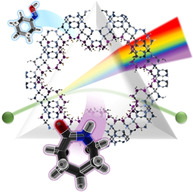

## Introduction

The emergence of hierarchical materials has led to an evolution in the design of catalysts for targeted chemical transformations. Taking inspiration from the hierarchical structuring that is prevalent in nature, basic units can be assembled into more complex constructs in order to modify bulk properties and develop advanced functionalities.^1^ Analogously, through synthetic manipulation it is possible to create porous materials with a network infrastructure that is interconnected on the micro‐ (<2 nm), meso‐ (2–50 nm) and/or macro‐ (>50 nm) scale.^2^ Hierarchically‐porous (HP) architectures are designed to retain the desirable physicochemical and catalytic properties of the microporous (MP) network, whilst relieving their diffusional constraints.^3^ In a well‐established field, the manipulation of framework topology is delivering novel, multimodal catalysts, and revealing new capabilities in traditional catalytic systems.^4^

The porous silicoaluminophosphates (SAPOs) appear prominently in the literature as solid‐acid catalysts. Starting from an electrovalently‐neutral aluminophosphate (AlPO) backbone, two mechanisms can lead to the incorporation of silicon dopant.^5^ In Type II substitution, Si^IV^ replaces P^V^, with an isolated Brønsted site formed by association with a charge‐balancing proton. Additionally, Type III substitution of both Al^III^ and P^V^ may accompany Type II, generating acidic aluminosilicate regions inside, or at their periphery of silicon islands.

Whether an AlPO tends to undergo Type II or Type III substitution by silicon is characteristic of the framework topology.^6^, ^7^ For example, at low Si loading, faujasitic SAPO‐37 (FAU)^5^ tends to incorporate Si via the Type II mechanism.^8^ In this case, it is possible to obtain a largely homogeneous distribution of isolated Brønsted acid sites^9^ and generate, in effect, a single‐site acid catalyst.^10^ This, however, has implications for the acid characteristics of the SAPO,^6^ since the isolated acid sites formed by Type II substitution are weaker than the aluminosilicate regions developed by Type III substitution.^11^, ^12^ Nonetheless, for certain applications the moderate acidity of SAPO‐37 has proven advantageous. Potter et al. have shown that MP SAPO‐37 can catalyze the liquid‐phase Beckmann rearrangement (BR) of cyclohexanone oxime in near‐quantitative yield.^13^ In this case, higher Si loadings proved detrimental to caprolactam selectivity, as the formation of silicon islands created strong acidity. Instead, high selectivity towards caprolactam was correlated with weaker acidity, and the presence of isolated Brønsted sites that accompany low Si‐loading. The same authors later investigated the activity of SAPO‐37 by studying the mobility of cyclohexanone oxime in FAU frameworks using quasi‐elastic neutron spectroscopy (QENS).^14^ At 373 K, it was found that the oxime travelled within SAPO‐37 by jump diffusion, with a jump distance of 4.5 Å (the separation between the faujasitic supercages). This diffusion behavior contrasted with the smooth, Fickian diffusion of cyclohexanone oxime sorbed inside the aluminosilicate zeolite‐Y (FAU) framework. The jump diffusion behavior was attributed to interaction between the oxime and the internal acid sites of SAPO‐37, characterized by inelastic neutron scattering experiments. The authors concluded that favorable interactions between cyclohexanone oxime and the Brønsted sites in SAPO‐37 were responsible for the high yield of caprolactam in the liquid‐phase BR.^14^

Thus, it is possible to appropriately balance the acid strength of SAPO‐37, such that it is strong enough to facilitate the transformation of oxime, and yet weak enough to release the lactam, once formed. Such tuneability offers immense catalytic potential but microporous materials, such as SAPO‐37, suffer mass transport constraints. Particularly in the industrial, vapor‐phase BR (for which weakly‐acidic catalysts have proven well‐suited)^15^ microporous materials can be disadvantaged by their susceptibility to coke‐induced deactivation. Thus, despite excellent initial activity, catalytic performance may progressively decline as carbonaceous deposits congest the micropores.

To circumvent this issue, we have used the synthetic technique of soft‐templating to prepare a HP SAPO‐37 catalyst with superior and sustainable activity under vapor‐phase conditions. More specifically, an organosilane mesoporogen^16^, ^17^, ^18^ has been used to template HP SAPO‐37 *for the first time*. Strategically, the organosilane has a hydrolysable trimethoxysilyl group that forms covalent bonds between organosilane molecules and the framework precursors, preventing expulsion of the micelles from the crystallizing microporous phase. On calcination, the organic component of the surfactant is eliminated, and a HP structure is formed, with siliceous species incorporated into the walls of the intracrystalline mesopores. As a result, the organosilane templating strategy not only has implications for pore architecture, but also the acid characteristics of a HP framework, since the mesopores are terminated with a high concentration of silanol groups (Si‐OH).^18^, ^19^, ^20^

To this end, a detailed physicochemical characterization has been undertaken to contrast the structure and interactions of hierarchical and microporous SAPO‐37. Probe‐based techniques in particular, have provided meaningful insight into the influence of the soft‐templating approach on framework architecture (volumetric analysis, PALS) and acid‐site strength (TPD, FTIR, NMR). These insights have provided a rationale by which to align the catalyst properties with the reaction conditions, leading to significantly enhanced catalytic outcomes in the BR.^15^

## Results and Discussion

### Structural Characterization

To prepare HP SAPO‐37, dimethyloctadecyl[3‐(trimethoxysilyl)propyl]ammonium chloride solution (the source of soft template) was introduced into the SAPO‐37 precursor gel in the final step prior to hydrothermal crystallization (Section SI.1). Elemental and thermogravimetric analyses (Sections SI.2 and SI.3) indicated that the organosilane was incorporated in the as‐synthesized HP SAPO‐37 material, and after calcination the HP framework was produced with a higher Si content than its MP analogue (Table S1). To ascertain that the crystalline, faujasitic structure of SAPO‐37 was retained after dual‐templating, the calcined products were analyzed by powder X‐ray diffraction (XRD). To the best of our knowledge, only one group has reported the synthesis of a hierarchical SAPO‐37 material.^21^ Although the authors identified mesoporosity by N_2_ gas adsorption and low‐angle XRD, the high‐angle XRD pattern of their MESO‐SAPO‐37 was markedly different from FAU,^22^ which suggested that the microporous framework was not crystallized.

Significantly, the XRD pattern of our organosilane‐templated SAPO (Figure S2) correlated with both MP SAPO‐37 and the FAU structure reported in the literature,^22^ indicating that phase‐pure HP SAPO‐37 was prepared with excellent retention of the faujasitic structure. Refinement of the cubic unit cell parameters revealed that the lattice dimensions of soft‐templated HP SAPO‐37 exceeded those of the MP analogue (Table S3), which was indicative of a mesopore‐induced unit cell expansion. Mesoporous structuring was also evidenced by a broad peak at 2*θ*≈2.5° in the low‐angle XRD spectrum of HP SAPO‐37^16^—a similar feature was not observed for the MP catalyst. Transmission electron microscopy (TEM) provided visual evidence of the mesoporosity in HP SAPO‐37 (Figure S3) as striations in the HP SAPO‐37 crystallites.^18^

Textural analysis of HP SAPO‐37 was also consistent with the presence of mesoporosity. As well as a hysteretic, Type IV N_2_‐gas adsorption isotherm, BJH analysis of HP SAPO‐37 (Figure S4) revealed a narrow peak in the mesopore range (≈2.6 nm) that, by its absence from MP SAPO‐37, was assigned to the organosilane‐templated mesopores.^18^ Whilst the surface area of HP SAPO‐37 was lower than MP SAPO‐37 (551 and 693 m^2^ g^−1^, respectively), this was attributed to the open FAU topology and the replacement of micropore surface by mesoporous void space. Otherwise, the quantifiable mesopore volume and large external surface area of HP SAPO‐37 were consistent with the presence of mesoporous architectures (Table S4).

### Positron annihilation lifetime spectroscopy

To complement the gas adsorption studies, the SAPO‐37 catalysts were analyzed by positron annihilation lifetime spectroscopy (PALS). A discussion of PALS theory can be found in Section SI.7.

**Figure 1:**
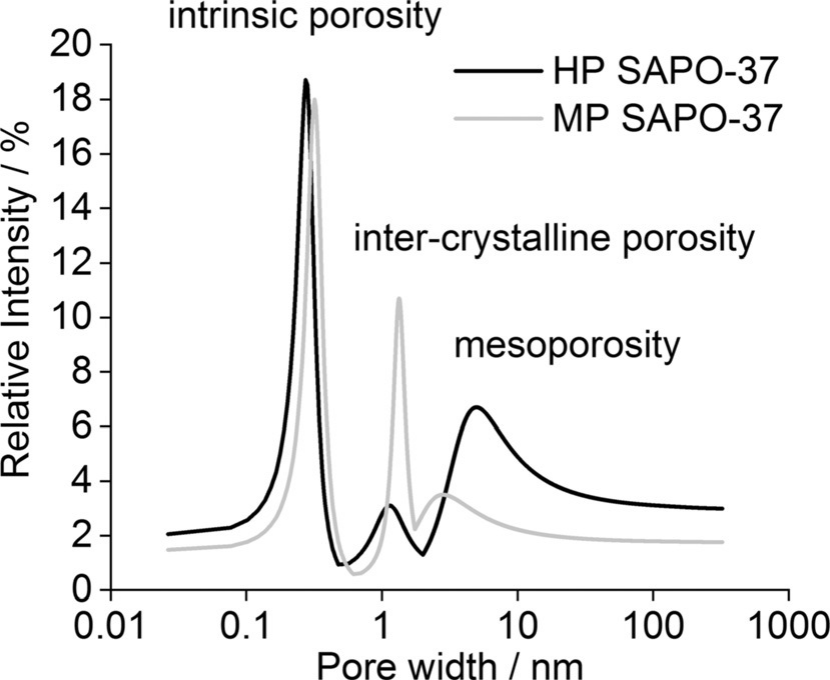
The pore‐size distribution of MP and HP SAPO‐37 obtained by PALS is a 2D representation of the pore distribution as derived from the PAScual software.^24^

The *ortho*‐positronium (*o*‐Ps) lifetimes in MP and HP SAPO‐37 were extracted from their respective PALS spectra by best fit to 5 components. The first component was fixed at 125 ps due to *para*‐positronium decay (annihilation of the positron and electron in a bound state of opposite spin), and the second at ≈400 ps due to free annihilation within the sample. The remaining three lifetimes (*τ*_3_, *τ*_4_, *τ*_5_) were attributed to *o*‐Ps decay, and thus identified a tri‐modal structure within the SAPOs. The results of the PALS data analysis are reported in Table 1, and the pore‐size distribution (PSD) is presented in Figure 1.

**Table 1:** The fitted PALS data for MP and HP SAPO‐37. The three lifetime components (*τ*) associated with o‐Ps annihilation in the catalyst pores are reported with their corresponding intensities (*I*). An average pore diameter (*d*) has been calculated from each lifetime component.

Sample	Lifetime [ns]			Intensity [%]			Average Pore Diameter [nm]		
	*τ*_3_	*τ*_4_	*τ*_5_	*I*_3_	*I*_4_	*I*_5_	*d*_3_^[a]^	*d*_4_^[b]^	*d*_5_^[b]^
MP SAPO‐37	0.965±0.085	8.470±0.405	33.1±4.9	18.0±4.2	10.7±0.5	3.5±0.5	0.32±0.03	1.34±0.03	2.75±0.30
HP SAPO‐37	0.848±0.024	5.891±0.232	61.2±3.0	18.7±1.9	3.1±0.1	6.7±0.2	0.28±0.01	1.13±0.02	5.08±0.34

[a] Lifetime converted into average pore diameter using the Tao‐Eldrup equation.^38^, ^39^ [b] Lifetime converted into average pore diameter using the rectangular Tao‐Eldrup equation.^40^

Unlike gaseous molecular probes, positrons do not rely on diffusion to gain entry to pores and cavities. Furthermore, the small dimensions of positronium (Bohr radius=0.53 Å) allow *o*‐Ps to occupy void spaces that are inaccessible to a larger, molecular probe.^23^ Consequently, a rationalization of the PALS data requires a consideration of the smallest pores and cavities in the sample. SAPO‐37 is a faujasitic framework, which comprises sodalite cages (or β‐cages) connected by double‐6‐rings to form a larger α‐cage. Some key dimensions associated with the α‐ and β‐cages are reported in Section SI.8.

The shortest *o*‐Ps lifetime component (*τ*_3_), yielded a void dimension (*d_3_*) of 0.28 nm for HP SAPO‐37, and 0.32 nm for MP SAPO‐37. Therefore, the *τ*_3_ component was associated with the intrinsic, zeolitic porosity (i.e. the structural microporosity) of the SAPO‐37 samples.^25^ Specifically, *d_3_* was attributed to *o*‐Ps annihilating within the smaller β‐cages and double six‐rings. Since the average diameter (*d*) and contribution (% *I*) of the *τ*_3_ component were comparable for HP and MP SAPO‐37, it was concluded that there was no significant difference in the intrinsic microporosity of the samples. In contrast, the *τ*_4_ and *τ*_5_ components differed quite significantly between the HP and MP catalysts. Average pore diameters (*d_4_*) of 1.34 and 1.13 nm were calculated from the *τ*_4_ components of MP and HP SAPO‐37, respectively, which were thus attributed to *o*‐Ps annihilating within the FAU supercage as well as a contribution from intercrystallite defects. Since the *τ*_4_ component made a significantly smaller contribution to the PSD of HP SAPO‐37 than MP SAPO‐37, this indicated that the drop in the number of crystallite defects within HP SAPO‐37 was likely due to a proportion of them merging with the larger mesopores.

An *o*‐Ps lifetime of *τ*>30 ns (as was observed for the *τ*_5_ component) is typically attributed to *o*‐Ps annihilating within mesopores.^26^ For MP SAPO‐37, the *d_5_* pore diameter (2.75 nm, due to *o*‐Ps annihilating within inter‐particle pores, in vacuum, or at defect sites)^26^ made relatively small contribution to the PSD. However, for HP SAPO‐37, the *d_5_* pore diameter was larger and more intense (*d_5_*=5.1 nm, *I_5_*=6.7 %), indicating significantly more mesoporosity than in MP SAPO‐37. Thus, the PALS analysis corroborated the N_2_ gas adsorption data, as both indicated that HP SAPO‐37 was more mesoporous than MP SAPO‐37.

### MAS NMR spectroscopy

**Figure 2:**
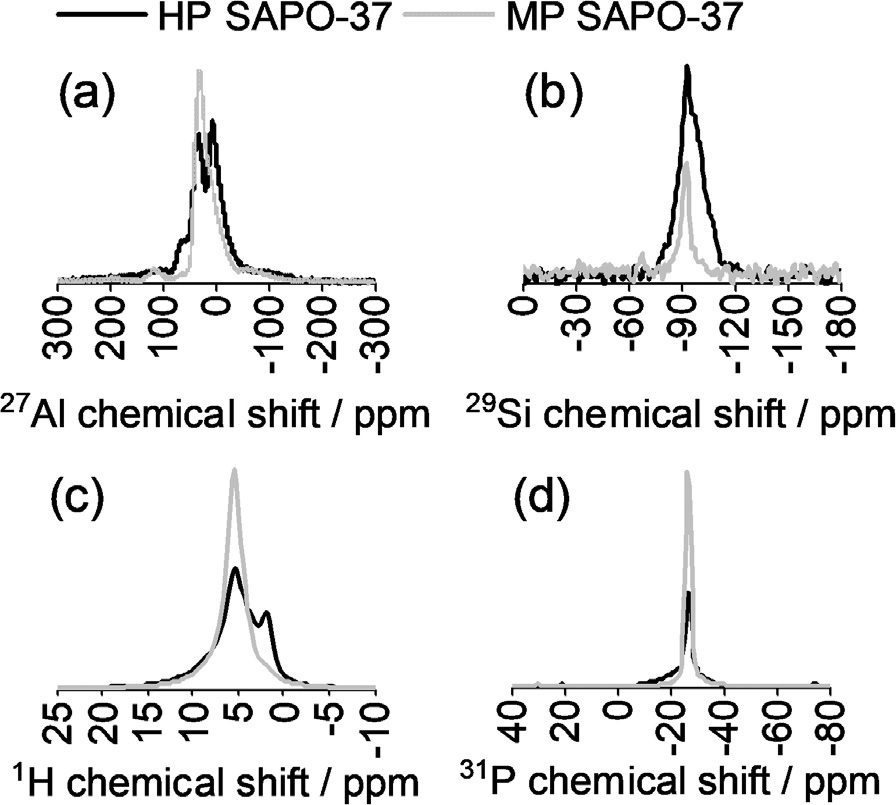
The a) ^27^Al, b) ^29^Si, c) ^1^H, and d) ^31^P MAS NMR spectra of HP and MP SAPO‐37 catalysts acquired at 400 MHz and 10 kHz spinning speed.

Magic‐angle spinning (MAS) NMR measurements were undertaken to probe the local coordination environment of the framework atoms in HP and MP SAPO‐37 (Figure 2).

The ^27^Al NMR spectrum of MP SAPO‐37 contained a single peak at 29.5 ppm, corresponding to Al in tetrahedral coordination [Al(4P)] within the framework.^27^, ^28^ However, the slight asymmetry of the ^27^Al resonance was indicative of a small number of 5‐ or 6‐coordinated aluminum defect sites in MP SAPO‐37.^29^ In HP SAPO‐37, two tetrahedral ^27^Al sites were identified: whilst the peak at 33.5 ppm was consistent with Al(4P) sites, the shoulder at 62.9 ppm evidenced zeolitic Al(4Si),^12^ originating from the mesopores. HP SAPO‐37 also contained a significant quantity of 5‐coordinated Al (6.3 ppm).^12^, ^30^ The more highly‐coordinated Al is a known artefact of the organosilane templating procedure^30^, ^31^, ^32^ and may be due to of extra‐framework Al species,^31^ an amorphous Al coordination environment,^12^ or hydrated tetrahedral Al sites.^17^

The ^29^Si MAS NMR of MP SAPO‐37 revealed a single Si environment at −92.1 ppm, corresponding to isolated Si(4Al9P) species in tetrahedral coordination within the framework.^33^ Implicitly, the Si(4Al9P) resonance also characterized bridging Brønsted acid sites in SAPO‐37, which accompany the (preferred)^8^, ^9^ Type II substitution of Si^IV^ for P^V^. The isolated, SAPO‐type Si sites were also prominent in the ^29^Si NMR of HP SAPO‐37, however significant intensity in the region up to −120 ppm identified the Si‐O‐Si bonding within the siliceous mesopores (derived from the organosilane template). In accord with the ^29^Si NMR data, MP SAPO‐37 exhibited a relatively well‐defined ^1^H proton resonance at 5.5 ppm, which was assigned to the bridging Si‐O(H)‐Al sites.^33^ Likewise, Brønsted protons were also present in HP SAPO‐37 (5.4 ppm), as well as silanol sites (1.9 ppm) from the organosilane‐templated mesopores.^34^, ^35^ Intensity in the region between the peaks of the Brønsted and silanol sites identified the protons associated with defects (P‐OH, Al‐OH), or aluminosilicate regions in the mesopores.^36^, ^37^

### Characterization of catalyst acidity

Since the activity of a catalyst for the BR is intrinsically linked to its acidity, an assessment of the nature, strength, and quantity of acid sites in the SAPO‐37 catalysts was fundamental for a rationalization of their performance in the BR. For this undertaking, the catalysts were analyzed in a series of probe‐based experiments, exploiting the interactions of basic molecules with the acid sites in the SAPO frameworks in order to study their acid characteristics.

### Temperature‐programmed desorption

**Figure 3:**
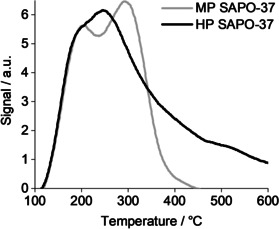
The NH_3_‐TPD profiles of MP and HP SAPO‐37 at a ramp rate of 10 °C min^−1^, after dosing with NH_3_ gas at 100 °C for 3 hours.

The temperature‐programmed desorption of ammonia (NH_3_‐TPD) from MP SAPO‐37 yielded a trace comprising two desorption peaks (Figure 3). The first peak, observed at 200 °C, was attributed to the loss of ammonia that was physisorbed within the SAPO pores, or weakly bound to defect sites (e.g. P‐OH, Al‐OH).^41^ Based on the ^1^H MAS NMR data, the second desorption peak of MP SAPO‐37 was assigned to the loss of ammonia from the more acidic, bridging Brønsted sites (from Type II substitution of P^V^ for Si^IV^).^13^, ^14^, ^42^

Although thermo‐desorption of ammonia from HP SAPO‐37 occurred with a single peak, it was evidently a convolution of multiple desorption processes. Notably, the desorption of ammonia from the bridging Brønsted sites occurred ≈45 °C lower in HP SAPO‐37 than MP SAPO‐37, which revealed that the framework acidity was moderated by the siliceous mesopore network.^43^, ^44^, ^45^ Nevertheless, HP SAPO‐37 also contained sites that were considerably more acidic than those found in MP SAPO‐37, as evidenced by the desorption peak in the region of 400 °C. The stronger acid sites may have arisen due structural distortions near to a Brønsted site, or due to aluminosilicate regions in the mesopores.^14^, ^17^

### ^15^N MAS NMR of ^15^N‐pyridine

**Figure 4:**
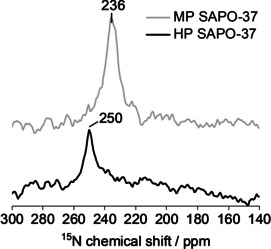
The ^15^N MAS NMR spectrum of ^15^N‐pyridine adsorbed in MP and HP SAPO‐37 reveals that hydrogen‐bonding interactions between the framework and probe were stronger in the MP catalyst. Data acquired at 600 MHz and 22 kHz spinning speed.

When ^15^N‐pyridine was adsorbed in HP and MP SAPO‐37, the ^15^N MAS NMR spectra both exhibited a single resonance (Figure 4), although the ^15^N chemical shift differed quite significantly between the two frameworks.

In HP SAPO‐37, the ^15^N peak at 250 ppm was consistent with pyridine hydrogen bonded to the weakly acidic silanols in the mesopores.^46^ Based on the relationship between ^15^N‐pyridine chemical shift and the strength of the ^15^N‐H interaction,^34^ the more upfield resonance observed with MP SAPO‐37 (236 ppm) was indicative of a stronger hydrogen bonding interaction with pyridine than that in HP SAPO‐37. Thus, the ^15^N NMR data were aligned with the NH_3_‐TPD analysis, as both characterized the predominance of stronger acidity in MP versus HP SAPO‐37.

**Figure 5:**
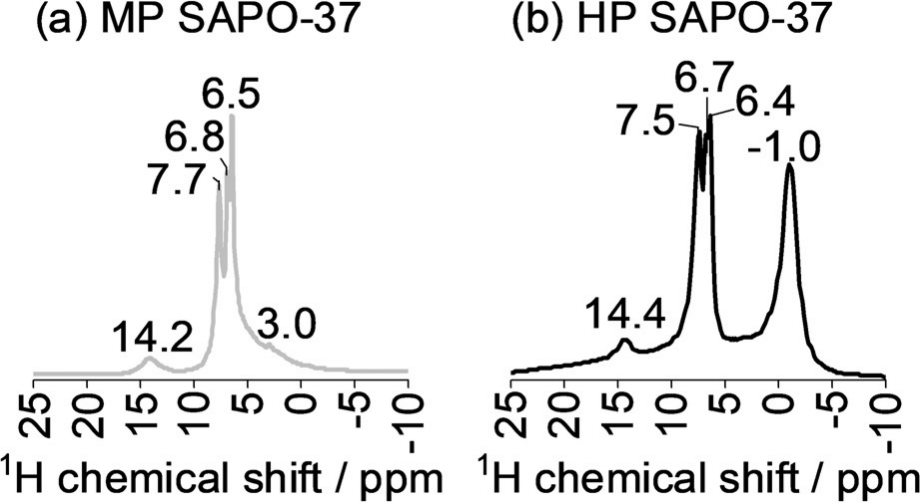
The ^1^H MAS NMR spectrum of ^15^N‐pyridine adsorbed in MP and HP SAPO‐37. Data acquired at 600 MHz and 22 kHz spinning speed.

The corresponding ^1^H MAS NMR spectra of the SAPO/^15^N‐pyridine samples are shown in Figure 5. In the 5–8 ppm region, peak intensity was attributed to a convolution of the pyridine ring‐protons (reported at 7.4–8.6 ppm)^47^ and framework Brønsted sites that are hydrogen‐bonded to pyridine (reported at ≈6 ppm),^48^ in accord with the ^15^N MAS NMR spectrum. Significantly, a peak at 14.2–14.4 ppm identified pyridinium species (typically 12–16 ppm)^48^, ^49^ in both HP and MP SAPO‐37. Only in the spectrum of MP SAPO‐37, was a small high‐field peak at 3.0 ppm resolved due to pyridine hydrogen‐bonded with Si−OH or Al−OH defect sites.^50^ The broad, high‐field peak at −1 ppm in the HP SAPO‐37 sample has not been reported previously. However, the negative ^1^H chemical shift indicated that a number of protons were very strongly shielded; this may be consistent with Si−OH⋅⋅⋅π interactions, if the pyridine molecules were oriented parallel to the surface of the mesopores.

### Probe‐FTIR

**Figure 6:**
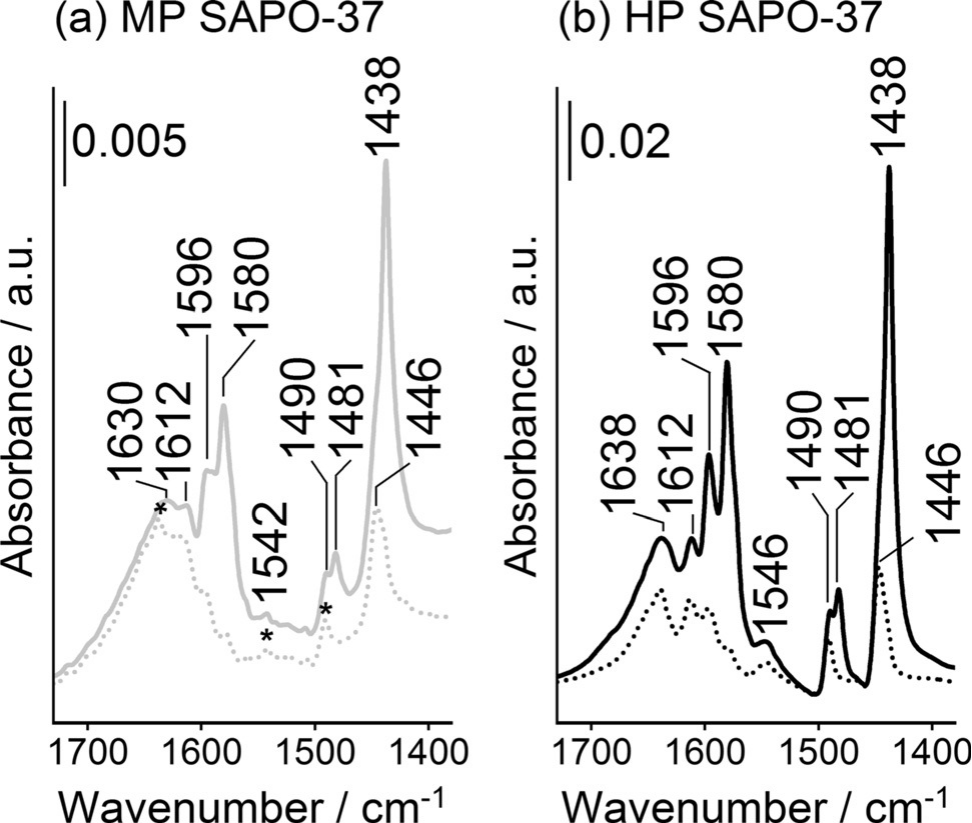
The FTIR difference spectra of a) MP SAPO‐37 and b) HP SAPO‐37 on exposure to pyridine vapor at room temperature (solid line) and after outgassing at room temperature (dotted line). * identifies peaks that are associated with the protonation of pyridine.

The adsorption of pyridine was also studied by FTIR spectroscopy. The FTIR spectra and peak assignments for pyridine adsorbed on MP and HP SAPO‐37 are reported in Figure 6 and Table S6, respectively.

A complex series of bands due to C−C aromatic ring vibrations are observed in the FTIR spectra of adsorbed pyridine on both HP and MP SAPO‐37. All pyridine modes were more intense for HP SAPO‐37 than MP SAPO‐37, revealing the higher adsorption capacity of the hierarchical framework. On removing the physisorbed pyridine at room temperature (bands at 1580, 1481 and 1438 cm^−1^), vibrations at 1612, 1596, 1446 cm^−1^ due to pyridine molecules hydrogen bonded to OH defects (Si−OH, P−OH or Al−OH)^51^ were visible, together with the bands at 1638, 1630, 1546, 1542, 1490 cm^−1^ due to protonated pyridine, consistent with the NMR data. The bands due to protonated species were comparable between the two catalysts, which confirmed that the micropores of SAPO‐37 (7.4 Å) were accessible to pyridine (kinetic diameter=5.4 Å).^52^ However, the slightly higher energy of the C−C ν_8a_ and ν_19b_ modes of protonated pyridine in HP SAPO‐37 (the most sensitive modes to interaction at nitrogen)^53^ alluded to the presence of the strongest acid sites identified by NH_3_‐TPD. The FTIR bands of coordinated pyridine on Lewis acid sites are not visible on SAPO‐37 catalysts, confirming the absence of Lewis acid sites, as also evidenced from the other characterization techniques.

**Figure 7:**
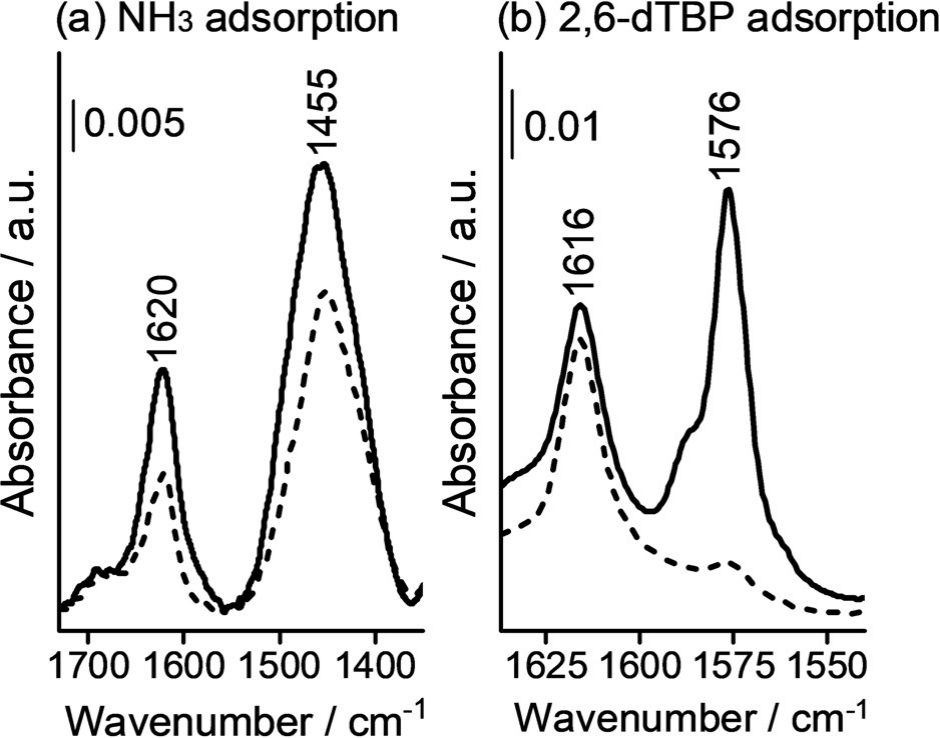
The FTIR difference spectra of HP SAPO‐37 on adsorption at room temperature of a) NH_3_ at 30 mbar and b) 2,6‐di‐*tert*‐butylpyridine at vapor pressure before (solid line) and after outgassing the probe molecules at room temperature (dotted line).

The probe‐based FTIR technique was also used to assess the accessibility of the Brønsted acid sites in HP SAPO‐37. First, ammonia was adsorbed to HP SAPO‐37 in order to quantify the total amount of Brønsted acid sites in the micropores and mesopores (Figure 7 a).

In the 1700–1300 cm^−1^ range, the FTIR spectra of NH_3_ adsorbed on HP SAPO‐37 exhibited two peaks. Of these peaks, the band centered at ≈1620 cm^−1^ was attributed to the ammonia molecules that were hydrogen bonded to Si‐OH groups, whilst the band centered at ≈1450 cm^−1^ identified the asymmetric bending mode (δ_asym_) of NH_4_^+^ species due to protonation of ammonia at stronger, Brønsted acid sites.^52^

Subsequently, the bulkier 2,6‐di‐*tert*‐butylpyridine (2,6‐dTBP) probe was adsorbed on HP SAPO‐37 (Figure 7 b). Due to steric hindrance (kinetic diameter=10.5 Å), 2,6‐dTBP can only interact with the most accessible acid sites, for example those located on the catalyst surface, or within the mesopores.^52^, ^54^, ^55^, ^56^ In the FTIR region associated with 2,6‐dTBP aromatic ring‐modes (1700‐1540 cm^−1^) two peaks were observed.^52^, ^54^, ^55^, ^56^ A higher energy band, centered at 1616 cm^−1^, characterized the protonated 2,6‐dTBPH^+^ species, and hence Brønsted acid sites. The ν_8a_ mode at 1576 cm^−1^ was attributed to weaker, hydrogen‐bonding interactions between 2,6‐dTBP and the Si‐OH or P‐OH sites in SAPO‐37 catalysts, which could be removed by outgassing at 298 K. The same probe molecules were also adsorbed on MP SAPO‐37 (Figure S6 and S7). Significantly, from the relative intensities of the characteristic modes, the FTIR spectra showed that considerably more 2,6‐dTBP was adsorbed in HP SAPO‐37 than MP SAPO‐37, demonstrating the improved accessibility of the HP framework via the mesopores.

The total number of accessible Brønsted acid sites (*N*) in HP SAPO‐37 was calculated from the ratio of the amount of adsorbed 2,6‐dTBP and NH_3_. *N* is reported in Table S7, with the nature and position of the IR bands of the corresponding protonated species, and also the accessibility factor (AF).^57^ Due to the significant steric bulk of the 2,6‐dTBP, the accessibility factor (AF=0.07 for HP SAPO‐37) reports the fraction of Brønsted sites on the external surface or within the mesopores.^55^, ^56^

Overall, probe‐based techniques have revealed some significant differences in the acid character of MP and HP SAPO‐37 catalysts. By NH_3_‐TPD, MP SAPO‐37 was found to contain a larger proportion of moderate‐to‐strong Brønsted acid sites than HP SAPO‐37, the acidity of the latter being moderated by the presence of weakly acidic silanols in the mesopores. Nevertheless, NH_3_‐TPD identified a number of very strong acid sites in HP SAPO‐37, which may have arisen due to structural distortions at the Brønsted sites and aluminosilicate regions associated with the mesopore network. The net stronger acidity of MP SAPO‐37 was evidenced by a larger upfield shift of the ^15^N MAS NMR resonance of adsorbed ^15^N‐pyridine, although both samples appeared only to engage in hydrogen‐bonding interactions with the probe. However, on inspecting the corresponding ^1^H MAS NMR, it was revealed that pyridine was not only hydrogen bonded to the framework, but also protonated at the Brønsted acid sites. The FTIR of adsorbed ammonia, pyridine, and 2,6‐dTBP identified both weak (Si‐OH, Al‐OH, P‐OH) and strong (Brønsted) acid sites in MP and HP SAPO‐37, but a larger amount of probe was adsorbed to HP SAPO‐37, which was consistent with the larger number of acid sites (i.e. silanol and Brønsted).^58^

### Catalysis

**Figure 8:**
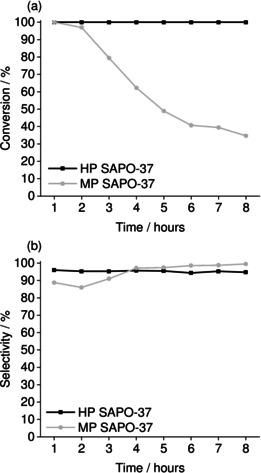
The a) conversion of cyclohexanone oxime and b) selectivity towards *ϵ*‐caprolactam in the vapor‐phase BR (300 °C, 0.79 h^−1^, oxime 10 g L^−1^ in ethanol) catalyzed by MP SAPO‐37 and HP SAPO‐37. By‐product is cyclohexanone.

Since the acid‐site characterization of HP SAPO‐37 had revealed the acidity of the SAPO‐37 framework was moderated by the presence of the weakly acidic silanols in the mesopores, the catalysts were tested in the vapor‐phase BR of cyclohexanone oxime at 300 °C (Figure 8). Previous studies have demonstrated that weak acid sites have greater propensity to enhance the yield of caprolactam in the vapor‐phase BR.^59^, ^60^

Under the conditions of the vapor‐phase BR, the difference in the catalytic activity of HP SAPO‐37 and MP SAPO‐37 was marked. Whilst HP SAPO‐37 sustained >99 % conversion of cyclohexanone oxime for 8 hours’ time‐on‐stream, conversion over MP SAPO‐37 declined progressively to 34.6 % in the same period. The loss of activity in MP SAPO‐37 was consistent with gradual blockage of the micropores due to accumulation of coke and nitrogenous residues,^61^ which would increasingly hinder access to the internal active sites. The observation that HP SAPO‐37 was resistant to deactivation implied that the active sites (whether in the micropores or mesopores) remained accessible with time‐on‐stream. We believe that the stronger acidity of MP SAPO‐37 may have contributed to its deactivation by promoting the accumulation of by‐products (high molecular weight condensation products) at Brønsted sites inside the micropores, since stronger acid catalysts tend to retain and re‐adsorb the lactam.^62^ Interestingly, the selectivity of MP SAPO‐37 increased slightly with time‐on‐stream, which suggested that some of the strongest and most unselective Brønsted acid sites were deactivated by coke deposition.^63^ Therefore, the enhanced catalytic performance of the HP SAPO‐37 can be attributed to the improved mass transport properties of the mesopores and the acidity‐moderating effect of its silanols.

High conversions in the Beckmann rearrangement is inherent to SAPO‐37 materials, as evidenced by the near‐quantitative yield obtained in the low temperature, liquid phase Beckmann rearrangement.^[s13]^ Furthermore, catalyst deactivation in the Beckmann rearrangement is typically associated with the formation of higher molecular weight condensation products in the gas‐phase. We have observed that the catalytic performance and stability of mesoporous MCM‐41^64^ is greatly impacted, due to formation of condensation products. Since the selectivity of HP SAPO‐37 was sustained at 100 %, there was no evidence to suggest that the catalyst would be deactivated.

To confirm that pore blockage was responsible for the deactivation of MP SAPO‐37 in the vapor‐phase BR, both catalysts were characterized post‐catalysis. Powder XRD confirmed that the FAU structure was retained in both HP and MP SAPO‐37 after catalysis, and thus framework collapse was excluded as the cause of MP SAPO‐37 deactivation (Figure S8a, Table S8). N_2_ adsorption experiments (Figure S8b) revealed that the surface area of HP SAPO‐37 was severely diminished post‐catalysis (from 551 to 141 m^2^ g^−1^), yet the mesopore volume (0.28 cm^3^ g^−1^) was essentially unchanged. However, at the end of the same 8‐hour BR reaction, MP SAPO‐37 was essentially non‐porous (BET surface area <10 m^2^ g^−1^). A micropore volume could not be calculated for either catalyst, which suggested that the micropores were inaccessible in MP and HP SAPO‐37. Nevertheless, the inaccessibility of the internal Brønsted sites of HP SAPO‐37 appeared to have no effect on its catalytic activity. Therefore, the BR in HP SAPO‐37 may have occurred predominantly at the less acidic (and hence more selective)^65^ silanol sites in the mesopores.

The post‐catalysis SAPOs were also subject to CHN (Table S9) and thermogravimetric analyses (Figure S9) in order to characterize the micropore blockage. CHN analysis of the post‐reaction catalysts revealed a larger deposit of carbonaceous and nitrogenous material in MP SAPO‐37 versus HP SAPO‐37, which correlated with the net stronger acidity of the MP framework.^60^ This observation was also supported by TGA, as a larger % weight loss occurred from MP than HP SAPO‐37. The % weight loss due to coke (Section SI.12) calculated for HP and MP SAPO‐37 was 16.5 and 23.3 %, respectively.

Thus, the TGA and CHN data indicated that the deactivation of MP SAPO‐37 was exacerbated by its tendency to accumulate coke deposits (likely due to its stronger acidity).^60^ Nonetheless, the fact that MP SAPO‐37 retained some activity, irrespective of the pore blockage, suggested that there was catalytic turnover at active sites on the surface of the SAPO‐37 crystallites. For HP SAPO‐37, the acidity‐moderating effect of the silanols in the mesopores was found to reduce the amount of coke that was accumulated within the framework, relative to MP SAPO‐37. Whilst TGA and CHN evidenced a significant accumulation of coke and nitrogenous residues in HP SAPO‐37, the mesopores conferred greater resistance to pore‐blockage, hence a sustained, high activity with time‐on‐stream.^63^

The HP SAPO‐37 catalyst was subsequently regenerated by calcination in air (16 hours, 550 °C). Encouragingly, when the recycled catalyst was tested in the vapor‐phase BR at 300 °C, it retained the performance of the fresh catalyst (Figure S10).

## Conclusion

HP SAPO‐37 was prepared via a “one‐pot” synthesis with an amphiphilic organosilane as the mesoporogen. Elemental (CHN) and thermogravimetric analyses indicated that the surfactant was successfully incorporated in the as‐synthesized product. Subsequent XRD analysis of the calcined HP SAPO‐37 revealed excellent retention of the crystalline FAU‐type structure, notwithstanding the presence of the mesoporous architectures (evidenced by low‐angle XRD, gas adsorption studies, and TEM imaging). Positron annihilation lifetime spectroscopy revealed the enhanced contribution of mesopores within the HP SAPO‐37 structure, in agreement with N_2_ gas sorption studies. These observations vindicated the organosilane‐template synthesis as an effective route to prepare HP SAPO‐37 with improved mass transport characteristics. To the best of our knowledge, these studies represent the only example of organosilane‐templated SAPO‐37, and the only example of hierarchically‐porous SAPO‐37 with a phase‐pure and crystalline FAU network in the bulk.

Probe‐based characterization (including TPD, FTIR, and MAS NMR) revealed the acidity‐moderating effect of the siliceous mesopores in HP SAPO‐37, and the improved accessibility of the Brønsted acid sites in the HP framework. In the vapor‐phase BR, the weaker acidity and mesoporosity of HP SAPO‐37 led to a marked enhancement in catalyst activity in the BR at 300 °C. The enhanced catalytic performance of the HP SAPO‐37 is consistent with a crystalline framework, verifying the efficacy of our synthetic strategy, as amorphous SAPO‐37 catalysts afford inferior catalytic efficiencies.^66^ Characterization of the post‐catalysis samples revealed a significantly larger carbonaceous/nitrogenous deposit in the MP SAPO‐37 than HP SAPO‐37, which was attributed to the increased formation of by‐products on the strong acid sites of MP SAPO‐37. Whilst coke deposition was observed on the HP SAPO‐37 catalyst some porosity was still retained, and the catalyst was effectively regenerated with no loss of activity or selectivity. Therefore, the role of the mesopores in the sustained performance of HP SAPO‐37 was two‐fold, since the weakly acidic silanol sites retarded coke deposition, and the larger pore volume of the mesopores were slower to be blocked by coke.

We believe this is the first example of using an organosilane‐mediated, soft templating method for creating a phase‐pure, highly crystalline, hierarchical faujasitic (FAU) zeotype with an enhanced contribution of mesopores in bulk, which affords enhanced mass‐transport characteristics and remarkable stability compared to its microporous counterpart, in solid‐acid catalyzed transformations. Our work seeks to highlight this unique, organosilane‐mediated synthetic approach as an effective design strategy for creating localized solid‐acid active centers to deliver novel, multimodal materials, and also to reveal new catalytic capabilities within traditional porous systems.

## Conflict of interest

The authors declare no conflict of interest.

## Supporting information

As a service to our authors and readers, this journal provides supporting information supplied by the authors. Such materials are peer reviewed and may be re‐organized for online delivery, but are not copy‐edited or typeset. Technical support issues arising from supporting information (other than missing files) should be addressed to the authors.

Supplementary
