# Supplementary material for: Probing the Design Rationale of a High‐Performing Faujasitic Zeotype Engineered to have Hierarchical Porosity and Moderated Acidity
Source: Angew Chem Int Ed Engl. 2020 Aug 25;59(44):19561–9. doi: 10.1002/anie.202005108 (PMC7692934; doi:10.1002/anie.202005108)
Supplement: Supplementary file 1 — Supplementary [file ANIE-59-19561-s001.pdf]

Angewandte Chemie
Eine Zeitschrift der Gesellschaft Deutscher Chemiker

Supporting Information

Probing the Design Rationale of a High-Performing Faujasitic Zeotype Engineered to have Hierarchical Porosity and Moderated Acidity

*Stephanie Chapman, Marina Carravetta, Ivana Miletto, Cara M. Doherty, Hannah Dixon, James D. Taylor, Enrica Gianotti, Jihong Yu, and Robert Raja**

anie_202005108_sm_miscellaneous_information.pdf

Table of Contents

Table of Contents 2
SI.1 Experimental Procedures 2
SI.2 Elemental analysis of the as-synthesized and calcined catalysts 4
SI.3 Thermogravimetric analysis of the as-synthesized and calcined catalysts 5
SI.4 Powder X-ray diffraction data 5
SI.5 TEM images of HP SAPO-37 6
SI.6 N_2 gas adsorption-desorption data 6
SI.7 PALS theory 7
SI.8 The structure of faujasite 8
SI.9 FTIR of adsorbed pyridine 9
SI.10 FTIR difference spectra of adsorbed NH_3 and 2,6-di-*tert*-butylpyridine on MP SAPO-37 and HP SAPO-37 9
SI.11 Characterization of the post-catalysis samples 11
SI.12 Analysis of TGA data 12
SI.13 HP SAPO-37 recycle test 13
SI.14 References 13
SI.15 Author Contributions 14

SI.1 Experimental Procedures

Catalyst Synthesis
The synthesis of HP SAPO-37 and MP SAPO-37 followed the same procedure, except that the latter did not include the addition of dimethyloctadecyl[(3-(trimethoxysilyl)propyl] ammonium chloride.

Solution A: Phosphoric acid (9.25 g, 85 wt. % in water, Sigma Aldrich) and deionized water (20 mL) were stirred together in a Teflon beaker, to which pseudo-boehmite (5.58 g, Condea Vista) was added over the course of 1 hour. Solution A was stirred for 7 hours.
Solution B: Tetra-n-propylammonium hydroxide (TPAOH, 38.69 g, 40 wt. % in water, Alfa Aesar), tetramethylammonium hydroxide pentahydrate (TMAOH, 0.37 g, Sigma Aldrich) and fumed silica (1.00 g, Sigma Aldrich) were stirred for 2 hours in a glass beaker. Solution B was added dropwise to Solution A and stirred for 68 hours. Dimethyloctadecyl[3-(trimethoxysilyl)propyl]ammonium chloride solution (DMOD, 3.43 mL, 42 wt. % in methanol) was added dropwise to the gel, which was stirred for 2 hours.
The gel was transferred to a Teflon-lined, stainless steel reactor and crystallized at 200 °C for 24 hours. The reactor was allowed to cool to ambient temperature before its contents were removed and distributed between 4 x 100 mL centrifuge tubes. The solid in each tube was washed 3 times with deionized water at 10,000 rpm, and then dried overnight in an oven at 80 °C. The solid (~ 32 g) was calcined in batches (~ 2 g) by heating to 550 °C (ramp rate of 2 °C min^{-1}) for 16 hours.

Characterisation

Powder X-ray diffraction patterns were acquired using a Bruker D2 diffractometer with Cu $K_{\alpha1}$ radiation. Unit cell refinements were performed using the CelRef software.[1] Low-angle X-ray diffraction patterns were obtained using a Rigaku SmartLab diffractometer with Cu rotating anode source.

Nitrogen adsorption measurements were performed at 77K using the Micromeritics Gemini 2375 Surface Area Analyser. Samples were degassed, under vacuum, at 120 °C for 12 hours prior to measurement.

For ICP-OES elemental analysis, samples were subject to acid digestion before analysis in the Varian Vista MPX CCD Simultaneous Axial ICP-OES.

NH_3-TPD measurements were performed using the Quantachrome Autosorb iQ-Chemi apparatus. Catalysts were pre-treated at 150 °C under a flow of helium gas for 2 hours. The samples were then dosed with ammonia gas for three hours at 100 °C, before desorption under a flow of helium gas, with a temperature ramp of 10 °C min^{-1} up to 600 °C.

Carbon, hydrogen, and nitrogen (CHN) elemental analysis was performed using the Thermo Carlo Erba Flash 2000 Elemental Analyser.

Thermogravimetric analysis was performed on the Netzsch TG 209 F1 Libra by heating under air from 30 - 900 °C at a ramp rate of 10 °C min^{-1}.

TEM images were acquired using the Hitachi HT7700 Transmission Electron Microscope with Morada G3 camera.

Positron annihilation lifetime spectroscopy (PALS)

The catalyst powders were packed to 2 mm either side of a Mylar sealed $^{22}NaCl$ positron source. The samples were measured at room temperature, under high vacuum (5 x 10^{-6} Torr) and placed between two EG&G Ortec spectrometers for a minimum of 4.5 x 10^6 integrated counts. The final spectra were analyzed using LT (Version 9.0) software[2] using a source correction (1.486 ns and 3.593 %). The spectra were best fitted to 5 components; the first component being fixed to 125 ps due to *para*-positronium decay, and the second at ~400 ps due to free annihilation within the sample. The remaining three lifetimes were attributed to *ortho*-positronium decay, and associated with a tri-modal pore structure within the materials. The zeolites featured intrinsic micro-porosity (τ_3) due to the porous zeolite cages and channels, the inter-particle micropores (τ_4) and larger mesopores (τ_5).[3] The lifetimes were converted to average pore diameters using the Tao-Eldrup equation for τ_3 and the Rectangular Tao-Eldrup equation for τ_4 and τ_5.[4-6] The pore-size distribution was calculated using PAScual (Version 1.4) software.[7]

MAS NMR characterization of the framework

The calcined catalyst was packed in a 4 mm zirconium oxide rotor before drying overnight in a fan-assisted oven at 100 °C. Whilst still hot, the rotors were sealed by capping with a turbine. All NMR experiments were performed at a sample rotation frequency of 11 kHz, on a 9.4 WB Ascend Bruker magnet with a Neo console, using a triple resonance 4 mm RevolutionNMR probe. The ^1H NMR spectra were referenced to adamantane at 1.8 ppm.[8] The ^{29}Si NMR spectra were referenced to tetrakis(thrimethylsiloxy)silane at -9.8 and -135.4 ppm.[8] The ^{31}P spectra were referenced to phosphoric acid (85 % in H_2O) at 0 ppm. The ^{27}Al spectra were referenced to $AlCl_3$ (1 M in H_2O) at 0 ppm. The ^{29}Si cross-polarization experiments were obtained with ramped cross polarisation[9] are the result of 24576 scans, with a contact time of 3 ms and spinal decoupling at 80 kHz during acquisition. The ^{31}P NMR experiments were recorded with direct excitation using 2 scans and 180 seconds between scans. Proton decoupling using SPINAL64 was applied during acquisition. The ^{27}Al experiments were recorded with one pulse excitation and no decoupling with 64 scans.

Probe-based MAS NMR with ^{15}N-pyridine

Calcined catalyst was transferred to a ceramic boat and sealed inside a Schlenk tube. The Schlenk tube was transferred to a furnace to dry under vacuum at 120 °C for 12 hours. After isolation from the vacuum, the Schlenk tube was cooled to room temperature and transferred to a glove back under N_2 atmosphere. 7 µL of ^{15}N-labelled pyridine (Sigma Aldrich) was added to the catalyst (0.07 g) with mechanical mixing. The catalyst-pyridine sample (0.02 g) was packed into a 3.2 mm thin wall pencil rotor. The experiments were performed on a narrow bore 14.1 T Agilent DDR2 spectrometer equipped with a 3.2mm triple resonance Agilent probe, at a spinning speed of 13 kHz. ^1H NMR spectra were referenced to adamantane at 1.8 ppm.[8] ^{15}N spectra were referenced to NH_4Cl at 39.3 ppm. The direct excitation was recorded with 300 scans, and a pulse delay of 300 s between scans.

Probe-based FTIR studies

FTIR spectra of self-supporting pellets were collected under vacuum conditions (residual pressure <10^{-5} mbar) using a Bruker Equinox 55 spectrometer equipped with a pyroelectric detector (DTGS type) with a resolution of 4 cm^{-1}. NH_3, pyridine, and 2,6-di-*tert*-butylpyridine were each adsorbed at room temperature using specially designed cells, permanently connected to a vacuum line for *in situ* adsorption–desorption measurements. FTIR spectra were normalized with respect the pellet weight and, whenever specified, are reported in difference-mode by subtracting the spectrum of the sample in vacuum, from the spectrum with adsorbed molecules. The total number of accessible Brønsted acid sites (N) was estimated using the Lambert–Beer law in the form $A = \varepsilon N \rho$, where A is the integrated area of the bands of the protonated species, ε is the molar extinction coefficient ($cm^2\ mmol^{-1}$), N is the concentration of the

vibrating species (mmol g^{-1}), and ρ is the density of the disk (mass/area ratio of the pellet, mg cm^{-2}). The accessibility factor (AF) is defined as the number of Brønsted sites detected by, 2,6-di-*tert*-butylpyridine adsorption, divided by the total number of Brønsted acid sites detected by ammonia adsorption.

Catalysis

Pelletised catalyst (0.2 g) was sandwiched between glass beads (1 mm diameter) within a cylindrical, quartz, fixed-bed reactor tube (4 mm diameter). The tube was transferred to within the heater unit of a flow-reactor setup, and the catalyst pre-treated by heating at 400 °C for 1 hour under a flow of He gas (50 mL min^{-1}). For catalysis, the temperature and He flow were reduced to 300 °C and 33.3 mL min^{-1}, respectively. A liquid-feed of 100 g L^{-1} of cyclohexanone oxime in ethanol was supplied to the reactor *via* electronic syringe pump, to maintain a weight hourly space velocity (WHSV) of 0.79 hr^{-1}. Simultaneously, an external standard feed of 100 g L^{-1} of mesitylene in ethanol was introduced into the exit feed at a WHSV of 0.79 h^{-1}. Once steady-state was established, samples were collected on an hourly basis, and analyzed using the Clarus 480 gas chromatograph with FID detector and Elite-5 column.

SI.2 Elemental analysis of the as-synthesized and calcined catalysts

Table S1. The synthesis gel ratio and empirical elemental composition of the calcined MP SAPO-37 and HP SAPO-37 catalysts.

Catalyst	Gel Composition	ICP-OES elemental analysis
MP SAPO-37	1 Al: 1.36 P: 0.21 Si : 0.95 TPAOH: 0.03 TMAOH : 30 H_2O	16.5 wt. % Al; 13.8 wt. % P; 4.0 wt. % Si
HP SAPO-37	1 Al: 1.36 P: 0.21 Si : 0.95 TPAOH: 0.03 TMAOH : 0.03 DMOD : 30 H_2O	21.5 wt. % Al; 10.7 wt. % P; 5.1 wt. % Si

Table S2. Carbon, hydrogen and nitrogen elemental analysis of HP and MP SAPO-37 catalysts as-synthesized, and after calcination in air at 550 °C for 16 hours.

**Catalyst**		**Elemental analysis**		
		**% C**	**% H**	**% N**
HP SAPO-37	as-synthesized	20.08	3.95	1.86
	calcined	0.17	2.29	0.00
MP SAPO-37	as-synthesized	20.45	4.49	1.98
	calcined	0.08	2.75	0.00

SI.3 Thermogravimetric analysis of the as-synthesized and calcined catalysts

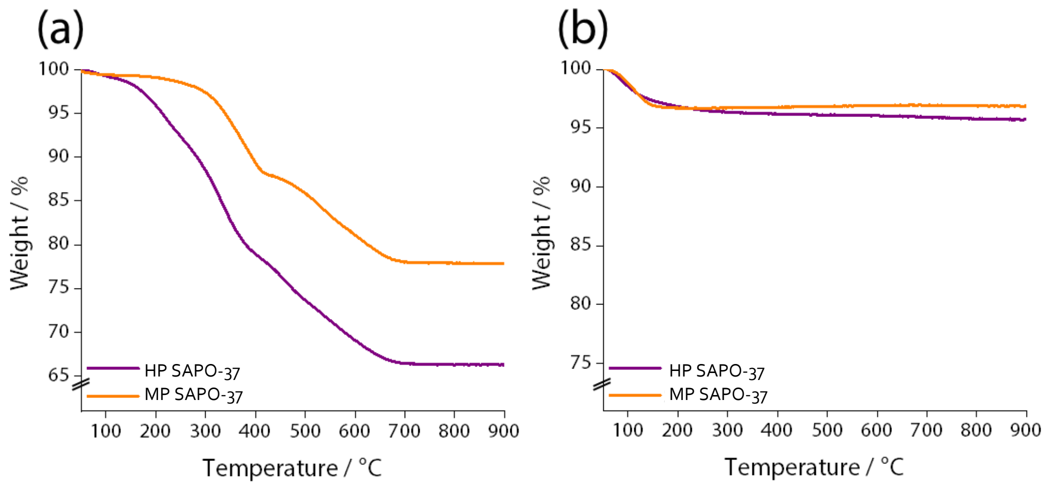

Figure S1. Thermogravimetric analysis of (a) as-synthesized and (b) calcined HP SAPO-37 and MP SAPO-37, acquired by heating in air from 30 - 900 °C at a ramp rate of 10 °C min^{-1}. Water desorption was observed at < 200 °C and, in the as-synthesized samples, weight loss at > 200 °C corresponded to the removal of organic templates.

SI.4 Powder X-ray diffraction data

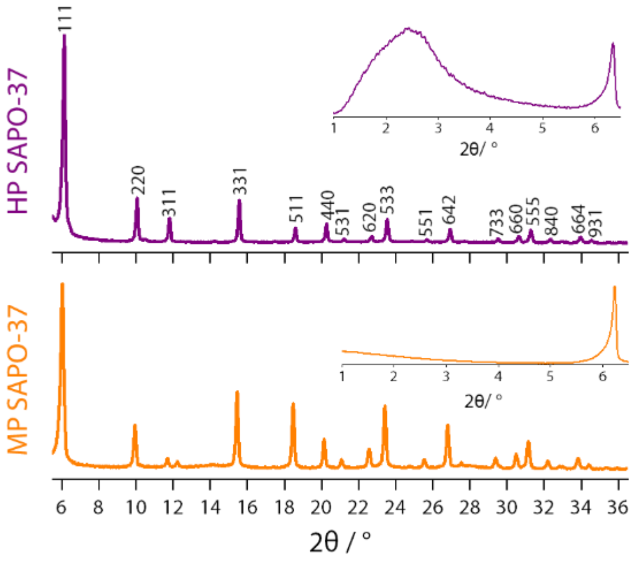

Figure S2. The indexed powder XRD patterns of HP SAPO-37 and MP SAPO-37 indicated that the catalysts were phase pure.

Table S3. The optimized unit cell parameters for MP SAPO-37 and HP SAPO-37 catalysts.

**Catalyst**	**Lattice Parameters**		**Space Group**	**Unit Cell Volume / Å^3**
	***a* = *b* = *c* / Å**	**α = β = γ / °**		
MP SAPO-37	24.46	90	Fd-3m	14628
HP SAPO-37	24.76	90	Fd-3m	15180

SI.5 TEM images of HP SAPO-37

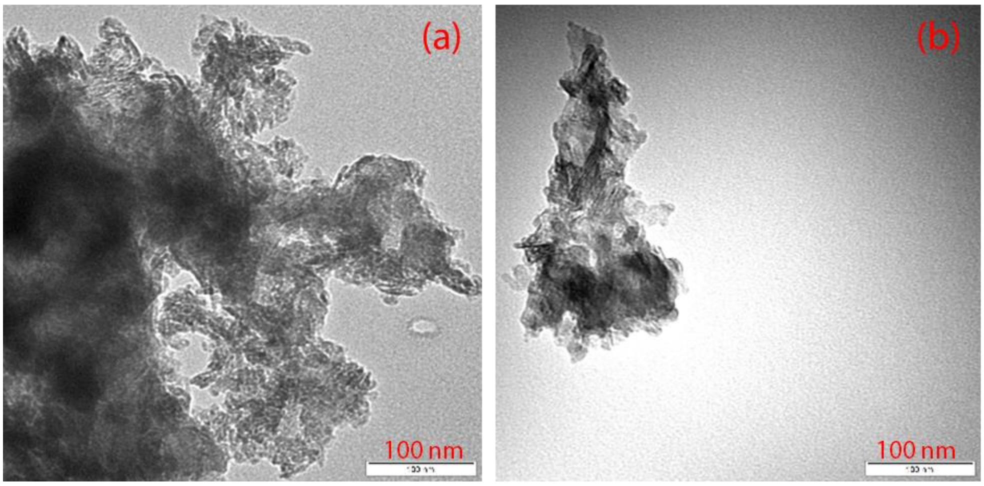

Figure S3. TEM images of HP SAPO-37 showing aggregated, rod-like crystallites that contain striations attributed to mesoporosity.

SI.6 N_2 gas adsorption-desorption data

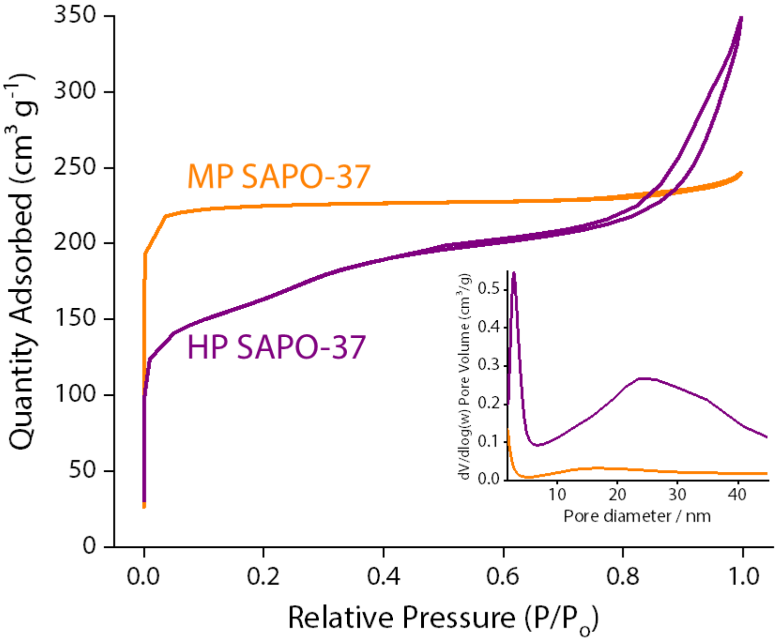

Figure S4. The N_2 gas adsorption-desorption isotherms at 77 K of MP SAPO-37 and HP SAPO-37, with their respective BJH adsorption pore-size distributions inset.

Table S4. The textural properties of MP SAPO-37 and HP SAPO-34 determined by N_2 adsorption-desorption studies.

**Catalyst**	**BET surface area / m^2 g^{-1}**	**Micropore volume / cm^3 g^{-1}**	**Mesopore volume / cm^3 g^{-1}**	**External surface area / m^2 g^{-1}**	**BJH mesopore diameter / Å**
MP SAPO-37	693	0.31	-	76	-
HP SAPO-37	551	0.12	0.29	230	26

SI.7 PALS theory

PALS is a non-destructive technique that monitors the lifetime of the *ortho*-positronium, *o*-Ps: a metastable system comprising of the bound state of a positron and an electron of the same spin. For textural characterization, PALS exploits the tendency of *o*-Ps to localize in electron-deficient defects (e.g. such as pores and voids) in an insulating material. The *o*-Ps will diffuse into a porous network and, where accessible, move into increasingly larger void spaces until annihilation.[10] If the *o*-Ps is not destroyed by pickoff annihilation with an electron from the surrounding matrix, it annihilates with an intrinsic vacuum lifetime of 142 ns (Figure S5).
Figure S6. Positron (e^+) formation and annihilation in a porous material. Positrons travelling in an insulating material may capture an electron to form the *o*-Ps species. *o*-Ps will tend to localize in the pores, travelling into increasingly larger voids (where accessible). The *o*-Ps may undergo pickoff annihilation with an electron (e^-) from the surrounding matrix, or escape to the surroundings and annihilate in vacuum. In either case, annihilation occurs with the production of gamma photons (γ).

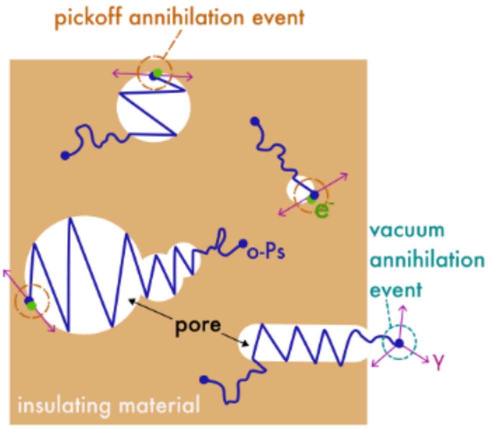

Therefore, any structural feature that increases the probability of pickoff annihilation (e.g. closed or narrow pores) will increase the probability of *o*-Ps annihilating with a lifetime < 142 ns. By applying suitable models,[4-6, 11] it is possible to relate the *o*-Ps lifetime to the size of the void in which it was annihilated. Significantly, the number of *o*-Ps annihilating at a particular lifetime is directly related to the contribution of each annihilation site; hence, the PALS intensity reflects the relative quantity and accessibility of the pores in a sample.

The *o*-Ps is sufficiently long-lived to quantify pore dimensions in the range 0.3 - 30 nm,[12] and the resolution of the technique is sufficient to discriminate different pore architectures within same pore-size regime.[13] Where PALS is able to distinguish and quantify porosity over multiple length scales, it has proven well suited to the study of hierarchical porosity.[3, 10, 12, 14-18] In particular, recent studies have sought to exploit the unique capabilities of the PALS technique to quantify the interconnectivity of micro- and meso-pores in hierarchical zeolites.[10, 12, 14-17] Ultimately, the *o*-Ps lifetime will tend towards the self-annihilation lifetime if the *o*-Ps can move into increasingly larger void spaces. Therefore, in a hierarchical system, the proportion of *o*-Ps annihilating in the micro- and meso-pores will depend on their relative pore volume and interconnectivity.[17]

SI.8 The structure of faujasite

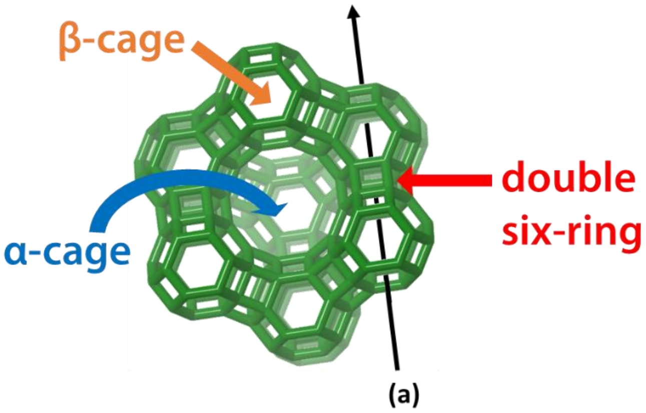

Figure S5. A section of the faujasite structure, indicating the sodalite (β-)cages that connect via double six-ring units to define a super-(α-)cage. The arrow marked (a) indicates the path of a diameter of a sphere < 2.53 Å within a channel formed by the double six-rings and the interior of the sodalite cages.

Table S5. The computed[9] diameter of the largest free-sphere that can diffuse through, and be included within the α- and β-cages of the faujasite framework.[19]

	**Structural component of the FAU framework**	
	**β-cage (sodalite)**	**α-cage**
**Maximum diameter of a sphere that can be included / Å**	6.23	11.24
**Maximum diameter of a sphere that can diffuse along/ Å**	2.53	7.35

SI.9 FTIR of adsorbed pyridine

Table S6. Assignment of the aromatic ring vibrations between 1400 - 1700 cm^{-1} in the FTIR spectra (Figure 10) of pyridine adsorbed on MP and HP SAPO-37.

Wavenumber / cm^{-1}	Sample		Assignment	Reference
	MP SAPO-37	HP SAPO-37		
1638	×	✓	ν_{8a} - protonated pyridine	20-21
1630	✓	×	ν_{8a} - protonated pyridine	21-22
1612	✓	✓	ν_{8a} – hydrogen-bonded pyridine	21
1596	✓	✓	ν_{8b} - hydrogen-bonded pyridine	20, 23
1580	✓	✓	ν_{8b} - physisorbed pyridine	20, 23
1546	×	✓	ν_{19b} - protonated pyridine	20-21
1542	✓	×	ν_{19b} - protonated pyridine	21
1490	✓	✓	ν_{19a} - protonated pyridine	20-21, 23
1481	✓	✓	ν_{19a} - physisorbed pyridine	23
1446	✓	✓	ν_{19b} - hydrogen-bonded pyridine	20, 23
1438	✓	✓	ν_{19b} - physisorbed pyridine	20-21, 23

SI.10 FTIR difference spectra of adsorbed NH_3 and 2,6-di-*tert*-butylpyridine on MP SAPO-37 and HP SAPO-37

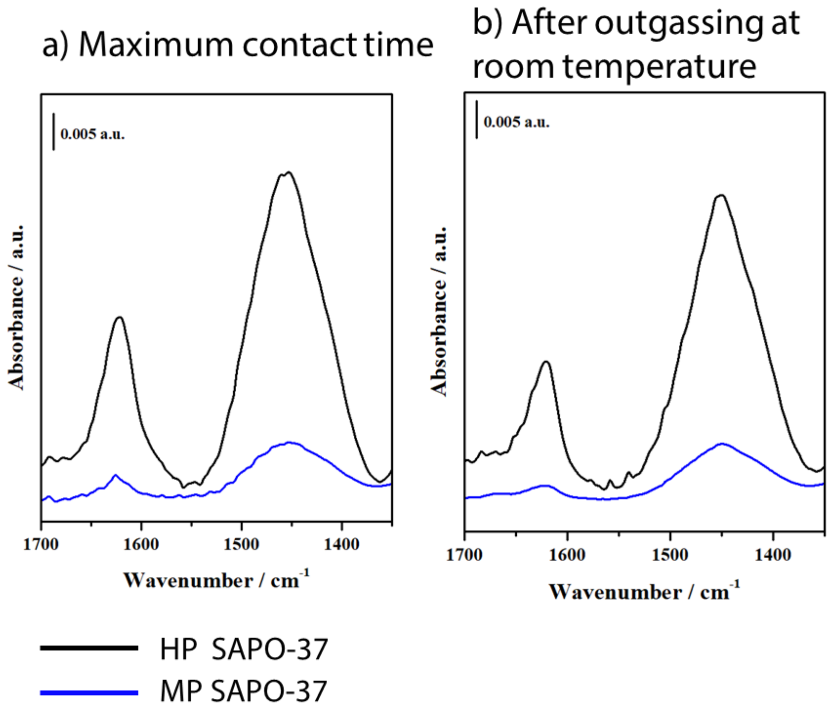

Figure S6. The FTIR difference spectra of MP SAPO-37 (blue) and HP SAPO-37 (black) on (a) adsorption of NH_3 at 30 mbar at room temperature and (b) after outgassing the probe molecules at room temperature.

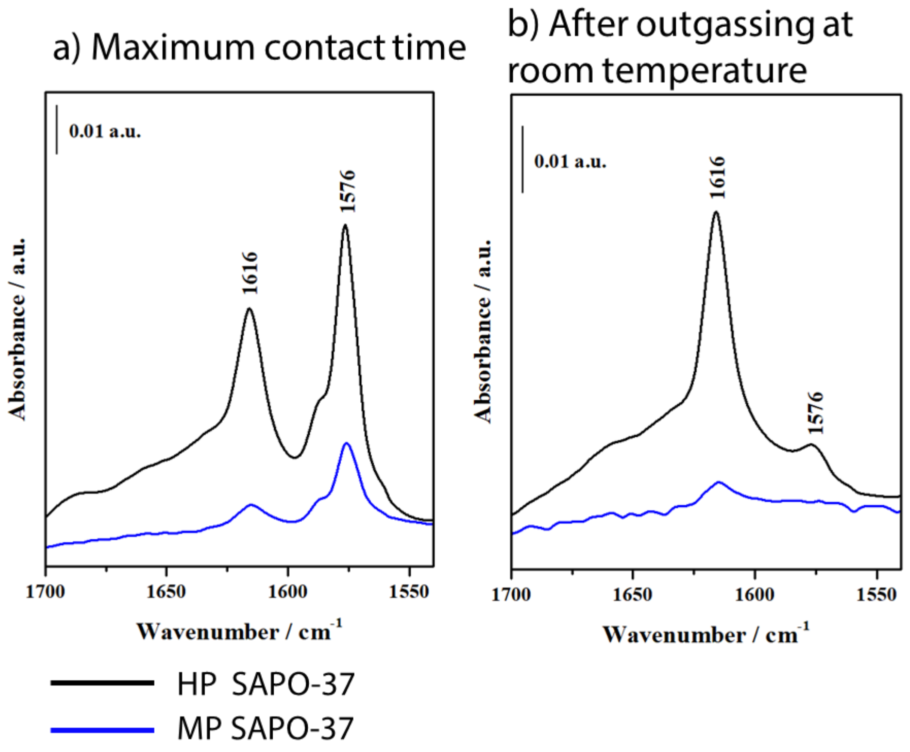

Figure. S7. FTIR difference spectra of MP SAPO-37 (blue) and HP SAPO-37 (black) on (a) adsorption of 2,6-di-tert-butylpyridine at room temperature and (b) after outgassing the probe molecules at room temperature.

Table S7. The concentration of accessible Brønsted acid sites (N) and the accessibility factor (AF) calculated from the ratio of the amount of 2,6-dTBP and NH_3 adsorbed to Brønsted acid sites in HP SAPO-37.

Probe species	IR mode	Position of IR mode / cm^{-1}	N / mmol g^{-1}	AF
NH_4^+	δ_{asym}	1450 [a]	19.0	1
2,6-dTBPH$^+$	ν_{8a}	1618 [b]	1.3	0.07

[a] ε = 0.147 cm^2 μmol^{-1} [24]

[b] ε = 0.5 cm^2 μmol^{-1} [25]

SI.11 Characterization of the post-catalysis samples

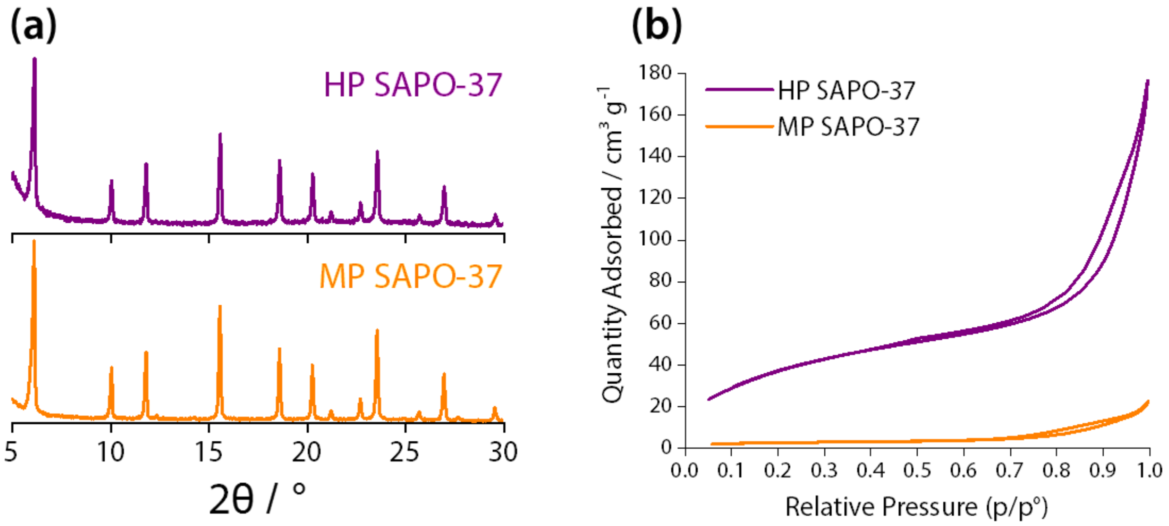

Figure S8. (a) The powder XRD pattern and (b) N_2 gas adsorption-desorption isotherm of HP SAPO-37 and MP SAPO-37 after 8 hours on-stream in the vapor-phase Beckmann rearrangement at 300 °C.

Table S8. The optimized unit cell parameters for MP SAPO-37 and HP SAPO-37 catalysts after 8 hours on-stream in the vapor-phase Beckmann rearrangement at 300 °C.

**Catalyst**	**Lattice Parameters**		**Space Group**	**Unit Cell Volume / Å³**
	***a* = *b* = *c* / Å**	**α = β = γ / °**		
MP SAPO-37	24.77	90	Fd-3m	15192
HP SAPO-37	24.76	90	Fd-3m	15175

Table S9. Carbon, hydrogen and nitrogen elemental analysis of HP and MP SAPO-37 after 8 hours on-stream in the vapor-phase Beckmann rearrangement at 300 °C.

Catalyst	Elemental analysis		
	% C	% H	% N
HP SAPO-37	11.73	1.05	1.75
MP SAPO-37	17.89	3.19	1.66

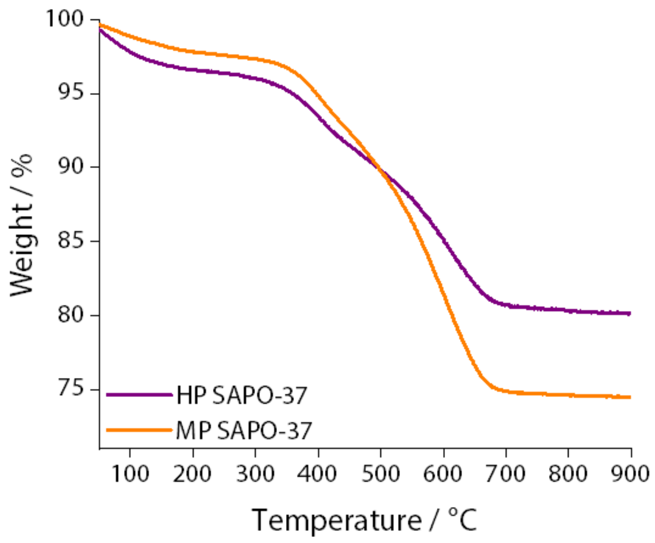

Figure S9. Thermogravimetric analysis of the HP SAPO-37 and MP SAPO-37 catalysts after 8 hours on-stream in the vapor-phase Beckmann rearrangement at a reaction temperature of 300 °C. TGA profile acquired by heating in air from 30 - 900 °C at a ramp rate of 10 °C min^{-1}. In the TGA trace, weight loss that occurred at temperatures < 200 °C was attributed to the desorption of water, whilst those at > 200 °C were assigned to the decomposition of nitrogenous and carbonaceous deposits from the vapor-phase reaction.

SI.12 Analysis of TGA data

A '% weight loss due to coke' ($wt.\ \%_{coke}$) was calculated for each catalyst using Equation 1, where $wt.\ \%_{200°C}$ and $wt.\ \%_{900°C}$ are the % weight of the sample after heating to 200 °C (i.e. after water loss) and 900 °C, respectively.

Equation 1: $$wt.\%_{coke} = wt.\%_{200\,°C} - wt.\%_{900\,°C}$$

HP SAPO-37: $wt.\ \%_{coke}$ = 96.6 % - 80.1 % = 16.5 %
MP SAPO-37: $wt.\ \%_{coke}$ = 97.8 % - 74.5 % = 23.3 %

SI.13 HP SAPO-37 recycle test

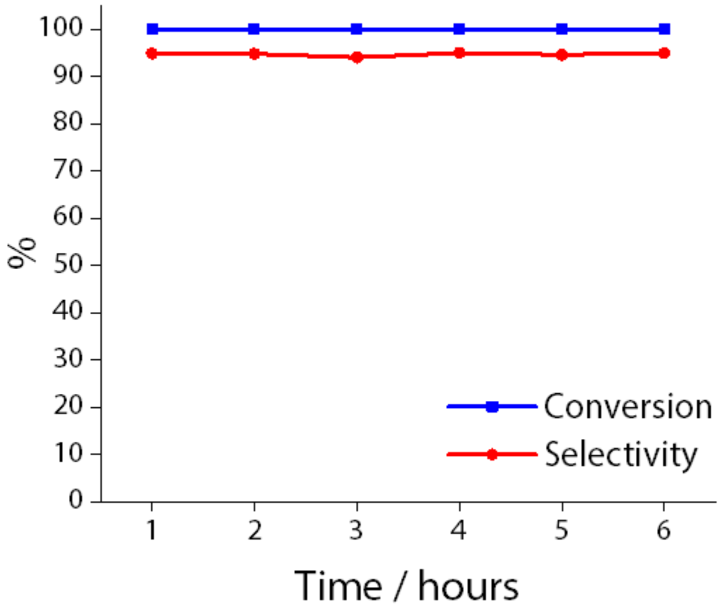

Figure S10. The conversion of cyclohexanone oxime (blue line), and selectivity towards ε-caprolactam (red line) in the vapor-phase Beckmann rearrangement (300 °C, 0.79 hr^{-1}, oxime 10 g L^{-1} in ethanol) catalyzed by HP SAPO-37 after recycle (air, 550 °C, 16 hours).

SI.14 References

[1] J. Laugier, B. Bochu, CelRef Version 3. http://www.ccp14.ac.uk/tutorial/lmgp/celref.htm.
[2] J. Kansy, *Nucl. Instrum. Methods Phys. Res. A* **1996,** *374*, 235-244.
[3] B. Zhu, L. Zou, C. M. Doherty, A. J. Hill, Y.S. Lin, X. Hu, H. Wang, M. Duke, *J. Mater. Chem.* **2010,** *20*, 4675-4683.
[4] T. L. Dull, W. E. Frieze, D. W. Gidley, J. N. Sun, A. F. Yee, A. *J. Phys. Chem. B* **2001,** *105*, 4657-4662.
[5] M. Eldrup, D. Lightbody, J. N. Sherwood, *Chem. Phys.* **1981,** *63*, 51-58.
[6] S. J. Tao, *J. Chem. Phys.* **1972,** *56*, 5499-5510.
[7] C. Pascual-Izarra, A. W. Dong, S. J. Pas, A. J. Hill, B. J. Boyd, C. J Drummond, *Nucl. Instrum. Methods Phys. Res. A* **2009,** *603*, 456-466.
[8] H. Shigenobu, H. Kikuko, *Bull. Chem. Soc. Jpn.* **1991,** *64*, 685-687.
[9] G. Metz, X. L. Wu, S. O. Smith, *J. Magn. Reson. A* **1994,** *110*, 219-227.
[10] A. Zubiaga, R. Warringham, M. Boltz, D. Cooke, P. Crivelli, D. Gidley, J. Pérez-Ramírez, S. Mitchell, *Phys. Chem. Chem. Phys.* **2016,** *18*, 9211-9219.
[11] K. Ito, H. Nakanishi, Y. Ujihira, *J. Phys. Chem. B* **1999,** *103*, 4555-4558.
[12] D. W. Gidley, H. -G. Peng, R. S. Vallery, *Ann. Rev. Mater. Res.* **2006,** *36*, 49-79.
[13] D. Dutta, S. Chatterjee, B. N. Ganguly, K. T. Pillai, *J. Appl. Phys.* **2005,** *98*, 033509.
[14] M. C. Duke, S. J. Pas, A. J. Hill, Y. S. Lin, J. C. D. d. Costa, *Adv. Funct. Mater.* **2008,** *18*, 3818-3826.
[15] J. Kenvin, S. Mitchell, M. Sterling, R. Warringham, T. C. Keller, P. Crivelli, J. Jagiello, J. Pérez-Ramírez, *Adv. Funct. Mater.* **2016,** *26*, 5621-5630.
[16] M. Milina, S. Mitchell, D. Cooke, P. Crivelli, J. Pérez-Ramírez, *Angew. Chem.* **2015,** *127*, 1611-1614.
[17] M. Milina, S. Mitchell, P. Crivelli, D. Cooke, J. Pérez-Ramírez, *Nat. Commun.* **2014,** *5*, 3922.
[18] J. Jagiello, M. Sterling, P. Eliášová, M. Opanasenko, A. Zukal, R. E. Morris, M. Navaro, A. Mayoral, P. Crivelli, R. Warringham, S. Mitchell, J. Pérez-Ramírez, J. Čejka, *Phys. Chem. Chem. Phys.* **2016,** *18*, 15269-15277.
[19] M. D. Foster, I. Rivin, M. M. J. Treacy, O. Delgado Friedrichs, *Micropor. Mesopor. Mater.* **2006,** *90*, 32-38.
[20] R. Buzzoni, S. Bordiga, G. Ricchiardi, C. Lamberti, A. Zecchina, G. Bellussi, *Langmuir* **1996,** *12*, 930-940.
[21] W. P. J. H. Jacobs, D. G. Demuth, S. A. Schunk, F. Schüth, *Micropor. Mater.* **1997,** *10*, 95-109.
[22] M. Akçay, *Appl. Catal. A: Gen.* **2005,** *294*, 156-160.
[23] I. Miletto, G. Paul, S. Chapman, G. Gatti, L. Marchese, R. Raja, E. Gianotti, *Chem. A Eur. J.* **2017,** *23*, 9952-9961.
[24] J. Datka, B. Gil, A. Kubacka, *Zeolites* **1995,** *15*, 501-506.
[25] K. Góra-Marek, K. Tarach, M. Choi, *J. Phys. Chem. C* **2014,** *118*, 12266-12274.

SI.15 Author Contributions

Stephanie Chapman (lead): catalyst synthesis and characterization, writing of original draft. Marina Carravetta (supporting): acquisition of NMR data. Ivana Miletto, Enrica Gianotti (supporting): acquisition and analysis of FTIR data. Cara M. Doherty (supporting): acquisition and analysis of PALS data. Hannah Dixon, James D. Taylor (supporting): acquisition of TPD data. Jihong Yu (supporting): insights on synthesis and facilitating structure-property links with hierarchical zeolites and zeotypes, Robert Raja* (supporting): conceiving original predictive design rationale, mapping catalytic descriptors with structure-activity analysis, validating outputs and coordinating research.
